# Interplay between Magnetism and Topology: Large Topological Hall Effect in an Antiferromagnetic Topological Insulator, EuCuAs

**DOI:** 10.1021/jacs.3c04249

**Published:** 2023-06-02

**Authors:** Subhajit Roychowdhury, Kartik Samanta, Premakumar Yanda, Bernard Malaman, Mengyu Yao, Walter Schnelle, Emmanuel Guilmeau, Procopios Constantinou, Sushmita Chandra, Horst Borrmann, Maia G. Vergniory, Vladimir Strocov, Chandra Shekhar, Claudia Felser

**Affiliations:** † Max Planck Institute for Chemical Physics of Solids, 01187 Dresden, Germany; ‡ Centre National de la Recherche Scientifique, Institut Jean Lamour, Université de Lorraine, Nancy 54011, France; § CRISMAT, CNRS, Normandie University, ENSICAEN, UNICAEN, 14000 Caen, France; ∥ Swiss Light Source, Paul Scherrer Institute, CH-5232 Villigen-PSI, Switzerland; ⊥ Donostia International Physics Center, 20018 Donostia-San Sebastian, Spain

## Abstract

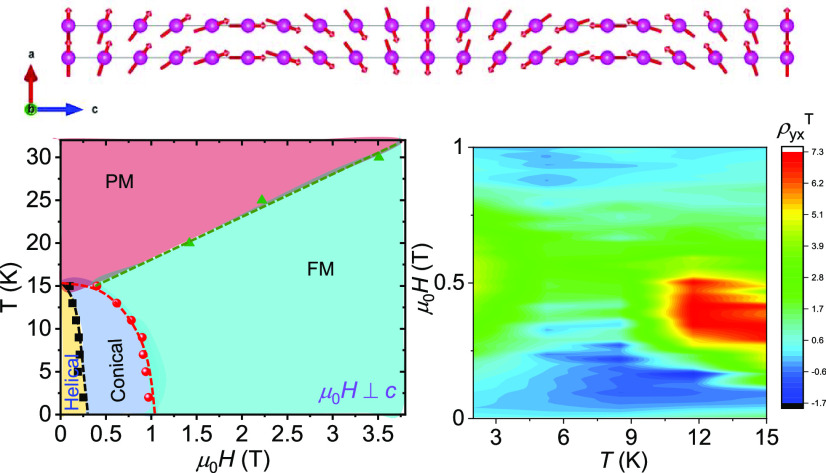

Magnetic interactions in combination with nontrivial band structures can give rise to several exotic physical properties such as a large anomalous Hall effect, the anomalous Nernst effect, and the topological Hall effect (THE). Antiferromagnetic (AFM) materials exhibit the THE due to the presence of nontrivial spin structures. EuCuAs crystallizes in a hexagonal structure with an AFM ground state (Néel temperature ∼ 16 K). In this work, we observe a large topological Hall resistivity of ∼7.4 μΩ-cm at 13 K which is significantly higher than the giant topological Hall effect of Gd_2_PdSi_3_ (∼3 μΩ-cm). Neutron diffraction experiments reveal that the spins form a transverse conical structure during the metamagnetic transition, resulting in the large THE. In addition, by controlling the magnetic ordering structure of EuCuAs with an external magnetic field, several fascinating topological states such as Dirac and Weyl semimetals have been revealed. These results suggest the possibility of spintronic devices based on antiferromagnets with tailored noncoplanar spin configurations.

## Introduction

Magnetic topological insulators (MTIs) are narrow bandgap semiconductors that combine magnetic order with nontrivial band topology and are expected to exhibit novel phenomena, such as the axion insulating state and the quantum anomalous Hall effect (QAHE), that have not been observed in their nonmagnetic counterparts.^1−5^ Currently, most magnetic topological insulators are fabricated by doping nonmagnetic topological insulators with 3d-ferromagnets.^6^ Unfortunately, fabrication, measurement, and property optimization of such devices are quite challenging, which limits the observation of important phenomena at low temperatures and/or high magnetic fields, thus limiting their usefulness in practical applications. Therefore, it is essential to search for intrinsic and stoichiometric MTIs with less rigid constraints to realize experimentally exotic quantum phenomena. In this scenario, intrinsic antiferromagnetic (AFM) topological insulators (TIs) with a -invariant provide a fertile ground for the exploration of such exotic quantum phenomena.^7^ In recent years, materials from the homologous series of the MnBi_2*n*_Te_3*n*+1_ family have been intensively studied as intrinsic and stoichiometric MTIs.^8−10^ An intrinsic van der Waals AFM with a nontrivial surface state was first identified in MnBi_2_Te_4_ by first-principles density functional theory (DFT) calculations and experiments.^8^ Recently, Deng et al. have reported a quantum anomalous Hall effect in MnBi_2_Te_4_ thin flakes at 1.4 K.^11^ However, the study of AFM TIs is still in its infancy.

As a result of nontrivial spin arrangements, MTIs exhibit a unique Hall effect with respect to their nonmagnetic counterparts.^12−15^ There is an additional contribution to the ordinary Hall effect in ferromagnetic (FM) systems, referred to as the anomalous Hall effect (AHE).^16^ In addition, a new additional Hall effect, namely, the topological Hall effect (THE), can also arise in materials with noncoplanar spin structures.^17,18^ In such a scenario, when conduction electrons pass localized spin moments, they can acquire a nonzero Berry phase with finite spin chirality, which acts as a magnetic field, resulting in the THE. Taking this scenario into account, the total Hall resistivity, ρ_yx_, can be expressed as ρ_yx_ = ρ_yx_^O^ + ρ_yx_^A^ + ρ_yx_^T^.^19^ Ordinary, anomalous, and topological Hall resistivities are denoted by the first, second, and third terms, respectively.

Initially, the THE was observed in noncentrosymmetric cubic phases of MnSi and FeGe with an extremely low topological Hall resistivity value (10^–3^–10^–2^ μΩ-cm).^20,21^ In recent years, the THE has been extensively observed in several systems such as frustrated magnets (Gd_2_PdSi_3_),^22^ Kagome lattices (YMn_6_Sn_6_ and Mn_3_Sn)^23,24^ and noncoplanar antiferromagnetic spin structures (MnP and Mn_5_Si_3_).^25,26^

Recently, europium (Eu)-based ternary compounds have attracted widespread attention due to their exciting magnetic properties resulting from Eu^2+^ (spin moment, *S* = 7/2) and exotic topological states, such as axion insulators (AFM-EuCd_2_As_2_ and EuIn_2_As_2_), an ideal Weyl semimetal (FM-EuCd_2_As_2_), Dirac surface states in EuSn_2_As_2_, and superconductivity in FM-EuFe_2_(As_0.7_P_0.3_)_2_.^27−31^

In the present study, we have performed detailed electrical and magnetic measurements on EuCuAs single crystals. Theoretical DFT calculations complemented by angle-resolved photoemission spectroscopy (ARPES) were used to study the electronic topological structure. We observed a large topological Hall (TH) resistivity with a maximum of ∼7.4 μΩ-cm at *T* = 13 K (below Néel temperature) due to the noncoplanar spin structure resulting from the metamagnetic transition when an out-of-plane magnetic field is applied. This result is further supported by neutron diffraction experiments. The observed TH value is significantly larger than those previously reported for frustrated magnets and noncoplanar magnetic structures. The analysis confirms that the ferromagnetic phase of EuCuAs contains only a single pair of Weyl points. Our results highlight the importance of the influence of magnetism on the band structure in realizing and even tailoring various unusual transport properties.

## Results and Discussion

**Figure 1:**
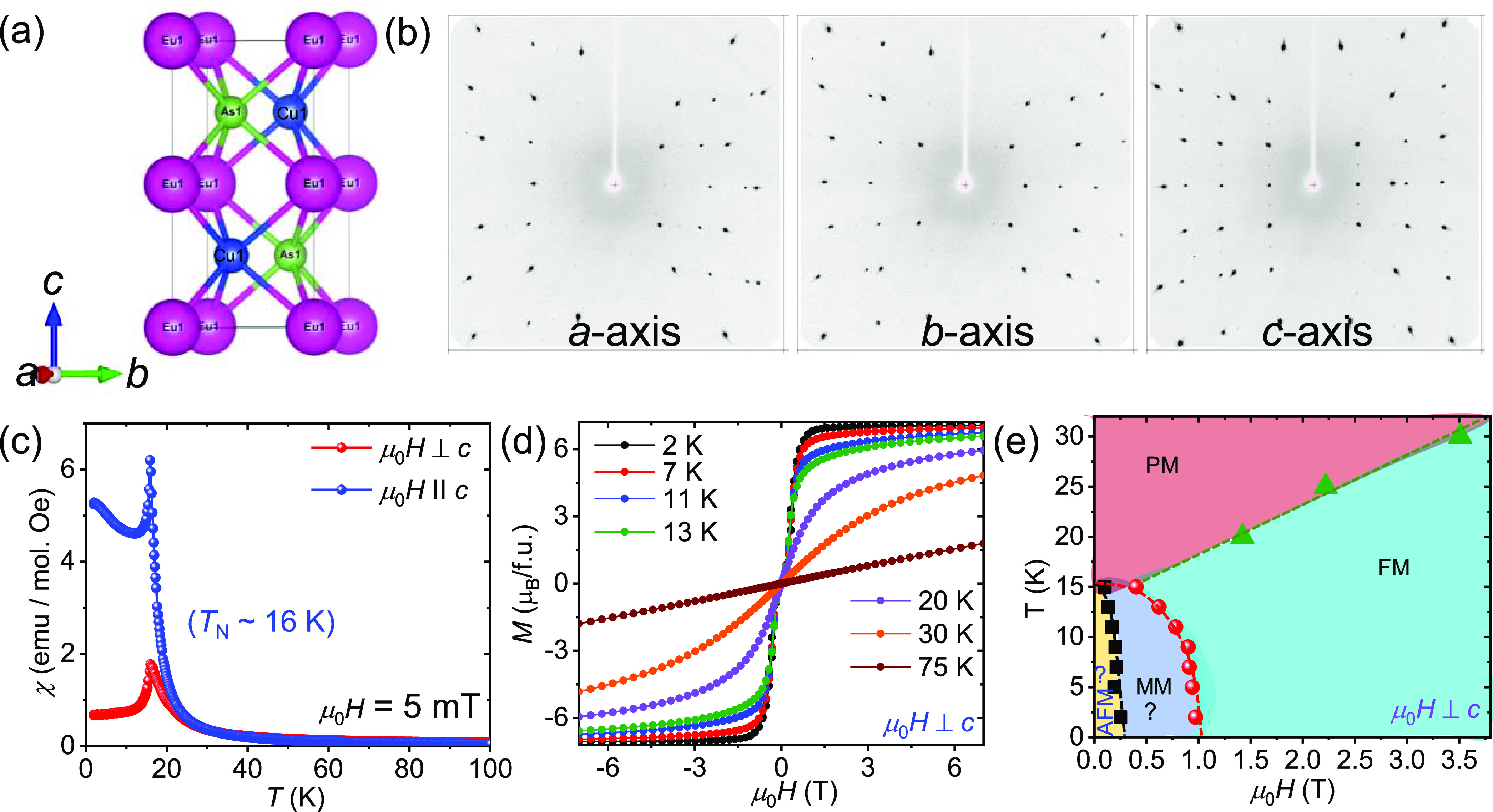
(a) Crystal structure of EuCuAs. Pink, blue, and green atoms represent Eu, Cu, and As, respectively. (b) Selected oscillation diffraction images of EuCuAs single crystals. (c) Temperature-dependent field-cooled magnetic susceptibility at μ_0_*H* = 5 mT for μ_0_*H* ⊥ *c* and μ_0_*H* || *c*. (d) Isothermal magnetization for μ_0_*H* ⊥ *c* at several temperatures. (e) Apparent magnetic phase diagrams for EuCuAs with μ_0_*H* ⊥ *c*. The symbols are extracted from the magnetization curves (c) and (d). Dashed lines guide the eye. AFM, MM, FM, and PM represent antiferromagnetic, metamagnetic, ferromagnetic, and paramagnetic states, respectively.

Small crystals were obtained by the method previously reported by Tong et al. using excess Sn as flux.^32^ However, using Bi flux (see Experimental Details, Supporting Information, SI), the typical size of the needle-shaped crystals was 3 × 0.5 × 0.2 mm^3^. EuCuAs crystallizes in a BeZrSi-type hexagonal layered structure (space group *P*6_3_/*mmc*) in which the Eu layers are separated by a Cu–As layer (Figure 1a). The structure can be represented as stacked planar honeycomb layers of Cu and As atoms along the *c*-direction (Figure S1, SI). In each layer, Cu and As occupy different sites. Eu is in the 2a Wyckoff position, while Cu and As are in the 2d and 2c Wyckoff positions, respectively. According to atomic distance considerations, the chemical bonding in the EuCuAs compound appears to be characterized by covalent bonding between Cu and As atoms (the Cu–As distance in EuCuAs (2.43 Å) is slightly larger than Pauling’s single bond radius of 2.31 Å for copper and arsenic) within the negatively polarized polyanions as well as electrostatic interactions with the positively polarized Eu atoms. It is possible to interpret the chemical bonding in EuCuAs compounds in terms of Zintl’s concept of electronic structure. Given that Eu is the most electropositive component, two valence electrons must be transferred to [CuAs]^2–^ in order to allow covalent bonding between the Cu and the As atoms. As shown by our ab initio calculations, the transition element (Cu) participates in the Cu–As bonding, which requires the consideration of the d-electrons.

It is noteworthy that EuCuAs can be derived from AlB_2_ (space group-*P*6/*mmm*) by applying minor distortions according to group-subgroup relations in the Bärnighausen formalism (the symmetry reduction is klassengleich).^33^ Thus, the group-subgroup scheme may provide future avenues for more topologically nontrivial materials with exotic physical properties by evaluating the symmetry reduction for the respective structures.

Scanning electron microscopy with an energy-dispersive X-ray analysis (EDAX) was used to evaluate the composition of the EuCuAs crystals (Table S1, SI). The single-crystal X-ray diffraction patterns of EuCuAs are shown in Figure 1b. The quality and orientation of the as-grown crystals were evaluated on a single-crystal diffractometer (Experimental section and Table S2, SI) using thin edge transmission. Unambiguous indexing revealed the expected hexagonal unit cell with lattice parameters *a* = 4.2598(3) Å and *c* = 8.2857(9) Å.^32^

The space group of EuCuAs contains the following symmetry operators: inversion symmetry(*I*), three-fold (*C*_3z_) and two-fold (*C*_2y_) rotation symmetry, twofold screw rotation symmetry (*S*_2z_ = {*C*_2z_|00}), and three mirror symmetries (*M*_z_, *M*_x_, and *M̂*_y_: *M̂*_y_ = {*M*_y_|00} is a glide mirror). We will discuss later the effect of the symmetry and magnetism, which play an important role in tailoring the different topological states in a single material according to our *ab initio* calculations.

To confirm the magnetic ordering temperature and behavior, we measured the field-cooled magnetic susceptibility with μ_0_*H* ⊥ *c* (χ_ab_) and μ_0_*H* || *c* (χ_c_) at a magnetic field of 5 mT (Figure 1c). At temperatures above 50 K, χ_ab_ and χ_c_ are nearly equal, and the material exhibits a nearly isotropic susceptibility, which is generally expected for the spin-only moment of the Eu^2+^ state. However, at low temperatures, there is a large magnetic anisotropy in the susceptibility. Both χ_ab_ and χ_c_ show a peak at ∼16 K, indicating an AFM transition at the Néel temperature, *T*_N_, similar to a previous report.^28^

**Figure 2:**
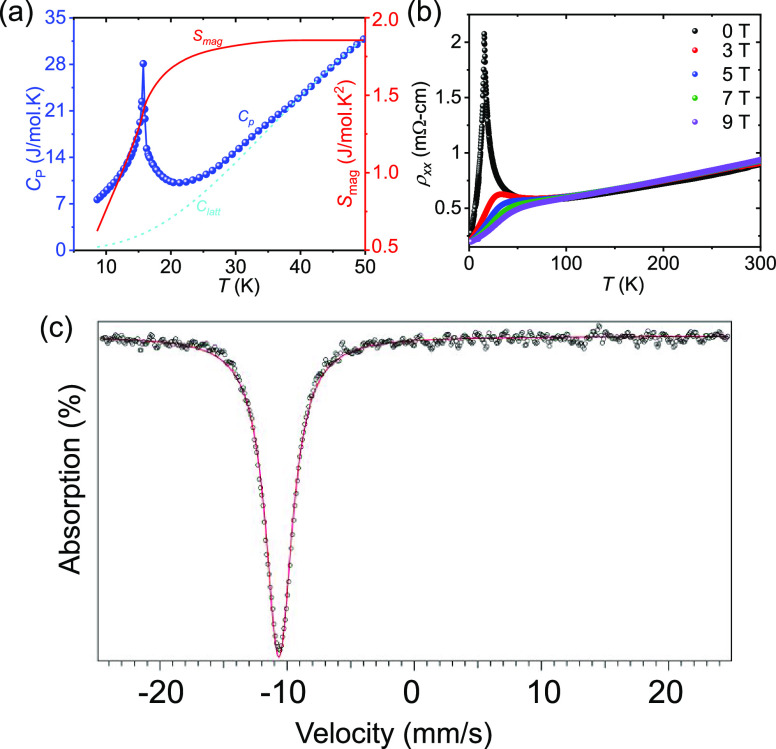
(a) Temperature-dependent heat capacity and magnetic entropy. (b) Variation of the resistivity, ρ_*xx*_, with temperature at different magnetic field strengths. (c) ^151^Eu Mössbauer spectra of EuCuAs at 300 K.

Magnetization isotherms with μ_0_*H* ⊥ *c* and μ_0_*H* || *c* are measured at selected temperatures ranging from 2 to 75 K (Figures 1d, S2, SI). Interestingly, above *T*_N_ (up to ∼30 K ∼ 2*T*_N_) we observe a large magnetization and nonlinear field dependence due to short-range ordering, which is also observed in the specific heat data (Figure 2a).

However, the nonlinearity in the magnetization is significantly enhanced at lower temperatures, *T* < *T*_N_. It can be seen that EuCuAs undergoes a first-order metamagnetic-like spin-flip transition with field hysteresis at *T* = 2 K (Figure 1d) when μ_0_*H* ⊥ *c*. The hysteresis starts at a low field of μ_0_*H* ≈ 0.25 T, rapidly enters the forced FM state, and saturates at ∼1 T (Figure 1e). A similar metamagnetic transition was previously observed earlier in MnBi_4_Te_7_ due to the noncollinear spin structure, which strongly influences the transport properties.^19^ At *T* = 2 K, the magnetization saturates at ∼7.04 μ_B_/f.u., which is close to the theoretical moment of Eu^2+^ ions. In contrast, no metamagnetic transition was obtained when μ_0_*H* || *c*. The magnetization increases linearly with the field and then saturates at ∼7.06 μ_B_/f.u. (Figure S2, SI).

The temperature dependence of the specific heat, *C*_p_(*T*), of the EuCuAs single crystal (Figure 2a) exhibits a sharp λ-type anomaly, indicating a magnetic order transition at *T*_N_ = 15.7(2) K. Due to the dominant magnetic contributions at low temperatures, electronic and phononic contributions in EuCuAs cannot be reliably analyzed as in other Eu compounds.^34,35^ An analysis of the magnetic specific heat *c*_mag_ = *c*_p_ – *c*_latt_, assuming a lattice contribution *c*_latt_ as shown by the dashed line in Figure 2a, yields a magnetic entropy *S*_mag_ = 1.9 J mol^–1^ K^–2^, which is in agreement with the expected value of *R*ln8. The short-range magnetic order contributions are prominent and visible up to temperatures above 2*T*_N_. Our room-temperature ^151^Eu Mössbauer experiment (Figure 2c) confirms the divalent nature of Eu, i.e., Eu^2+^, as previously also observed in EuMn_2_P_2_.^36^

The temperature-dependent longitudinal resistivity ρ_xx_ of EuCuAs is shown in Figure 2b. From 300 to 50 K, ρ_xx_ decreases linearly with decreasing temperature, indicating a metallic nature. Typically, the ρ_xx_ value is ∼0.27 mΩ-cm at 2 K and increases to 0.90 mΩ-cm at 300 K, i.e., the residual resistivity ratio [RRR = ρ_xx_ (300 K)/ρ_xx_ (2 K)] value for EuCuAs is 3.33, illustrating the high-quality single crystal. When the temperature is further decreased below 50 K, ρ_xx_ increases and then decreases after the Néel temperature is reached. This abrupt increase can be attributed to the increase in scattering due to the long-range critical fluctuations of the magnetic spins around *T*_N_, while the decrease after *T*_N_ can be attributed to the emergence of a long-range ordered state of the Eu moments. The magnetic ordering peak is dramatically reduced with the magnetic field, resulting in negative magnetoresistance (MR).

**Figure 3:**
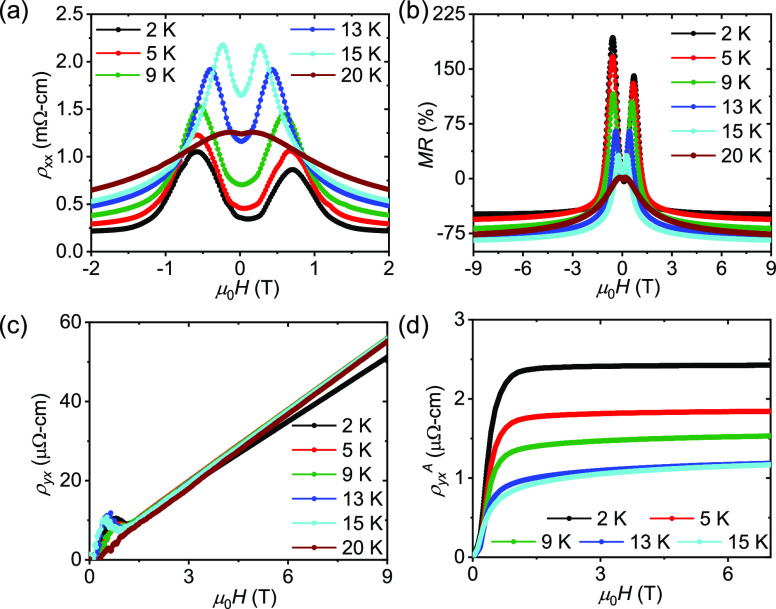
Field-dependent (a) resistivity, ρ_xx_, (b) transverse magnetoresistance, (c) Hall resistivity, ρ_yx_, and (d) anomalous Hall resistivity, ρ_yx_^A^ of EuCuAs at different temperatures.

To understand the role of magnetism in this material, we measured both the longitudinal and Hall resistivities of the EuCuAs crystal. It is possible to observe the AFM–FM spin-flip transitions from the field-dependent resistivity plots at different temperatures, where magnetic fields are applied along the out-of-plane, and electric current flows parallel to the *c*-axis (Figure 3a). Figure 3b shows the transverse magnetoresistance (MR = (ρ_xx_(μ_0_*H*) – ρ_xx_(0)/ρ_xx_(0)). The MR curve of EuCuAs is closely related to the magnetic state of the system. In the low-field region below 15 K, the observed positive MR value increases as the applied field increases to its maximum value near the spin-flip transition field. At higher magnetic fields, the MR continues to decrease and finally becomes negative. The MR plots show that the peak moves toward lower fields and decreases in intensity with increasing temperature, finally disappearing above *T*_N_. These properties can be attributed to a metamagnetic phase transition, which is consistent with our magnetization results. A maximum negative MR of 85% is obtained at 15 K, close to the Néel temperature. The spin disorder is strongest at temperatures around *T*_N_, and the associated carrier scattering is most effectively suppressed in this region, leading to the enormous negative MR.

Figure 3c shows the field-dependent Hall resistivity (ρ_yx_) at different temperatures. Above the magnetic transition temperature (*T* > 20 K), ρ_yx_ becomes linear up to 9 T, suggesting the applicability of a single carrier model in EuCuAs. The carrier (hole) concentration of our sample is ∼1.1 × 10^20^ cm^–3^ at 50 K. At *T* < *T*_N_, a hump-like anomaly appears in the low-field region.

However, such a feature is not observed in the *M* vs μ_0_*H* plot (Figure 1d). The evidence strongly suggests the existence of another type of Hall effect known as the topological Hall effect (THE), in addition to the ordinary Hall effect. Now, ρ_yx_ can be expressed as ρ_yx_ = *R*_0_μ_0_*H* + *R*_S_*M* + ρ_yx_^T^, where the first, second, and third contributions denote the ordinary, anomalous, and topological Hall resistivities, respectively.^19^ The resulting anomalous Hall resistivity (ρ_yx_^A^) is shown in Figure 3d. A maximum ρ_yx_^A^ of ∼2.5 μΩ-cm is observed at 2 K, and the corresponding anomalous Hall conductivity, AHC (σ_xy_^A^ ≈ ρ_yx_^A^/ρ_xx_^2^) is ∼60 Ω^–1^ cm^–1^ (Figure S3, SI).

**Figure 4:**
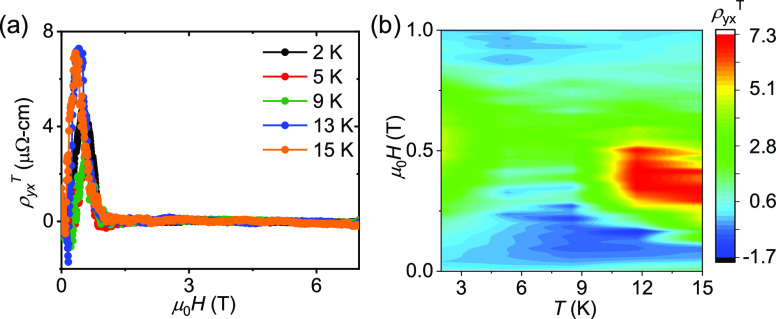
(a) Field-dependent topological Hall resistivity, ρ_yx_^T^. (b) Topological Hall resistivity as a function of temperature and magnetic field in a contour plot.

By subtracting the ordinary Hall resistivity and ρ_yx_^A^ from the total resistivity, we obtain the values of ρ_yx_^T^ at different temperatures, as shown in Figure 4a. To visualize the variation of the THE, the μ_0_*H* – *T* phase diagram is shown in Figure 4b as a contour plot by extracting the ρ_yx_^T^ values over the measured temperature range. Interestingly, a large maximum value of ρ_yx_^T^ of ∼7.4 μΩ-cm is observed at *T* = 13 K. This value is even higher than the “*giant topological Hall effect*” of Gd_2_PdSi_3_ (∼3 μΩ-cm), suggesting that EuCuAs is a suitable material for spintronics.^22^

**Figure 5:**
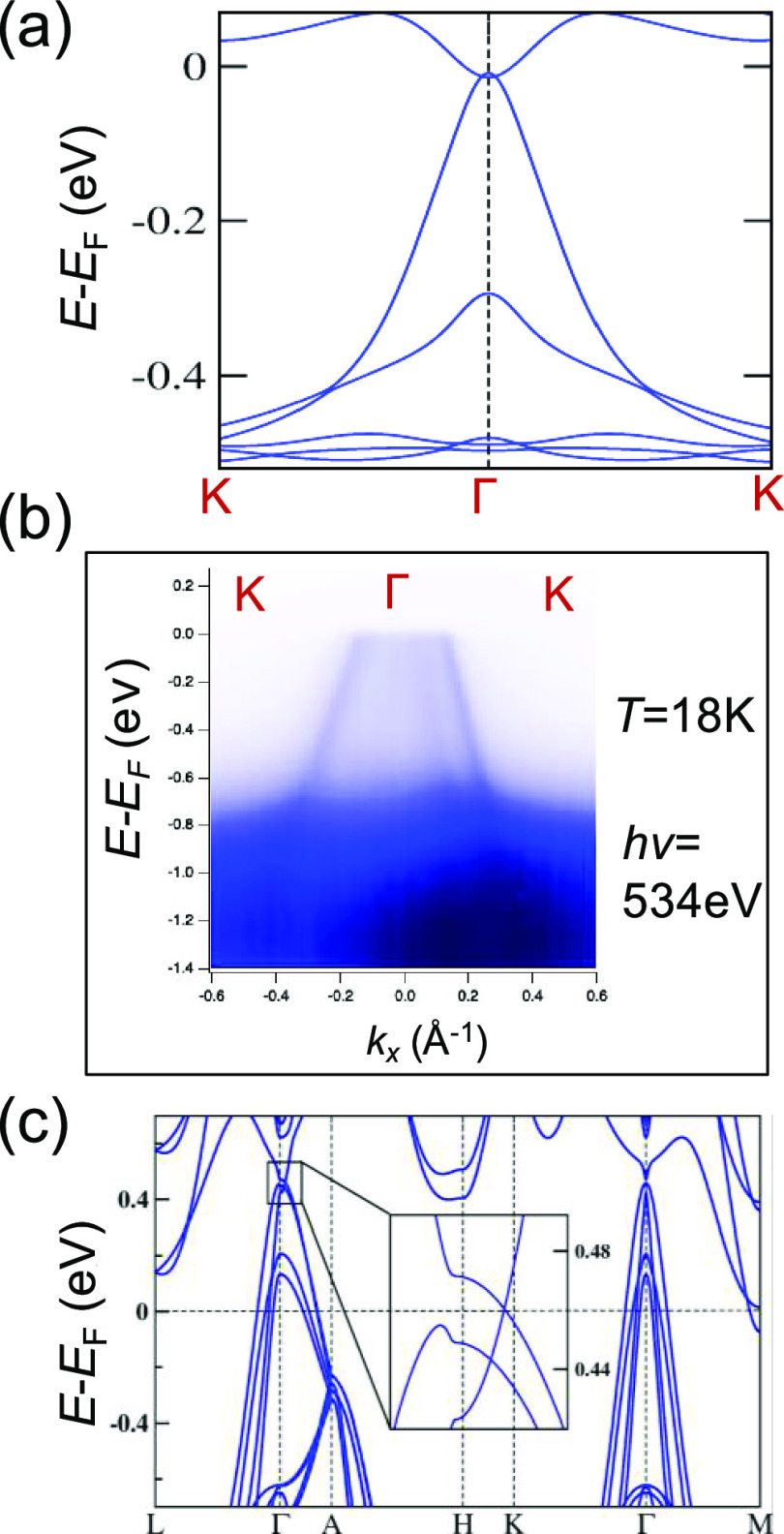
(a) Band structure of the paramagnetic phase of EuCuAs along the *K*–Γ–*K* path. (b) ARPES intensity plot along the Γ–*K* direction measured at 18 K. (b) Band structure of the ferromagnetic phase of EuCuAs.

To understand the correlation between the different magnetic spin states and the induced topological states in EuCuAs, we performed detailed first-principles DFT calculations, which also helped to explain our electrical transport data as well (Figures 5, S4–6, SI). Here, we calculate the band structures of three different magnetic states, namely the paramagnetic (PM), AFM, and FM states of EuCuAs. We observe a localized magnetic moment of 6.9 μ_B_ at the Eu 4f site, corresponding to half-filled states (*S* = 7/2). Due to the half-filled *f*-orbitals, the orbital magnetic moment in the ground state is completely quenched and finally equal to zero.

Above *T*_N_ ≈ 16 K, the system is PM. The band structure of the PM phase, calculated using a combination of the generalized gradient approximation (GGA), Coulomb interaction strength (Hubbard *U*), and spin-orbit coupling (SOC), is shown in Figures 5a and S6, SI. Importantly, we observed a crossing point between the valence and conduction bands near the Fermi level on the *Γ*-*A* path, called the Dirac point with fourfold degeneracy due to the preservation of both inversion and time-reversal symmetry (Figures S4a and S6, SI). Around the Fermi energy, we found a semimetallic ground state dominated by the Cu-3d, Cu-4s, and As-4p states.

We calculated both the in-plane and out-of-plane AFM spin configurations. For the antiferromagnetic AFM-*x* phase of EuCuAs when the Néel vector is oriented along the *x*-direction, the generator of the magnetic space group is *Cmcm*. Furthermore, in the AFM-*x* phase, the rotational symmetry along the *z*-direction is broken in addition to the time-reversal symmetry. Therefore, the Dirac point is unstable. As a result, the valence and conduction bands detach, resulting in a band gap of ∼190 meV, while the band inversion is maintained. Thus, the AFM state of EuCuAs is also topologically nontrivial. Analysis of the orbital character shows that the bands near the Fermi energy are dominated by Cu-3d, Cu-4s, and As-4p states. Due to the correlation effect, the fully spin-polarized 4f orbitals of each Eu are pushed down to >1 eV below the Fermi level.

To understand the origin of the AHC in the FM phase of EuCuAs, we also calculated the band structure of the FM configuration also in which the 4f spin of Eu is aligned along the *c*-axis (Figure 5c). Consequently, the degeneracy of the band is lifted, and we observe two Weyl points along the Γ–*A* path. Similar Weyl points were previously realized only in FM-EuCd_2_As_2_.^29^ However, in the present case, the Weyl points are far from the Fermi level. Furthermore, we calculated the AHE near *E*_F_ using a tight-binding Hamiltonian model with maximally localized Wannier functions of the Eu s, d, f; Cu s, p, d; and As p states. The calculated AHC value for the FM phase is qualitatively close to our experimentally observed value, suggesting that the intrinsic Berry curvature contributes significantly to the AHC in FM-EuCuAs (Figure S5, SI). Based on the first-principles calculations and symmetry/topology analysis, we show that rich magnetic topological states can be realized in EuCuAs depending on the magnetic configurations.

To investigate the experimental electronic structure, we performed synchrotron-based ARPES spectra of EuCuAs single crystals at 18 K (i.e., paramagnetic region) along the *K*–Γ–*K* direction with *h*ν = 534 eV (Figure 5b). The band dispersion of PM-EuCuAs was calculated using DFT and shows reasonable agreement with the ARPES measurements (Figure 5a,b). The Dirac point was estimated to be a few hundred meV above the Fermi level. In the future, the Fermi level could be raised to be closer to the Dirac point by electron doping and/or gating.

Noncollinear spin structures in antiferromagnets are likely to have a substantial THE.^37^ As a result of symmetry breaking coupled with significant SOC, such materials produce a net Berry curvature in momentum space and an inherent AHE. From our DFT calculations, it can be concluded that the AHC in FM-EuCuAs is mainly governed by the Berry curvature. Due to the separation of the two magnetic hexagonal layers of Eu by a nonmagnetic layer, there is a competition between AFM and FM coupling in EuCuAs. This reduces the interlayer AFM exchange coupling. A key factor influencing the transition from the A-type AFM state to the FM state is the strength of the interlayer exchange coupling (*J*) and the uniaxial anisotropy (*K*).^38^ A similar observation was previously reported in MnBi_4_Te_7_, where the relative strengths of *J* and *K* result in a canted state with a large THE.^19^ Due to the parallel projection of spins in a magnetic field, the THE is suppressed as the magnetic field increases. The large topological Hall resistivity value can be attributed to the noncollinear spin structure of EuCuAs.

**Figure 6:**
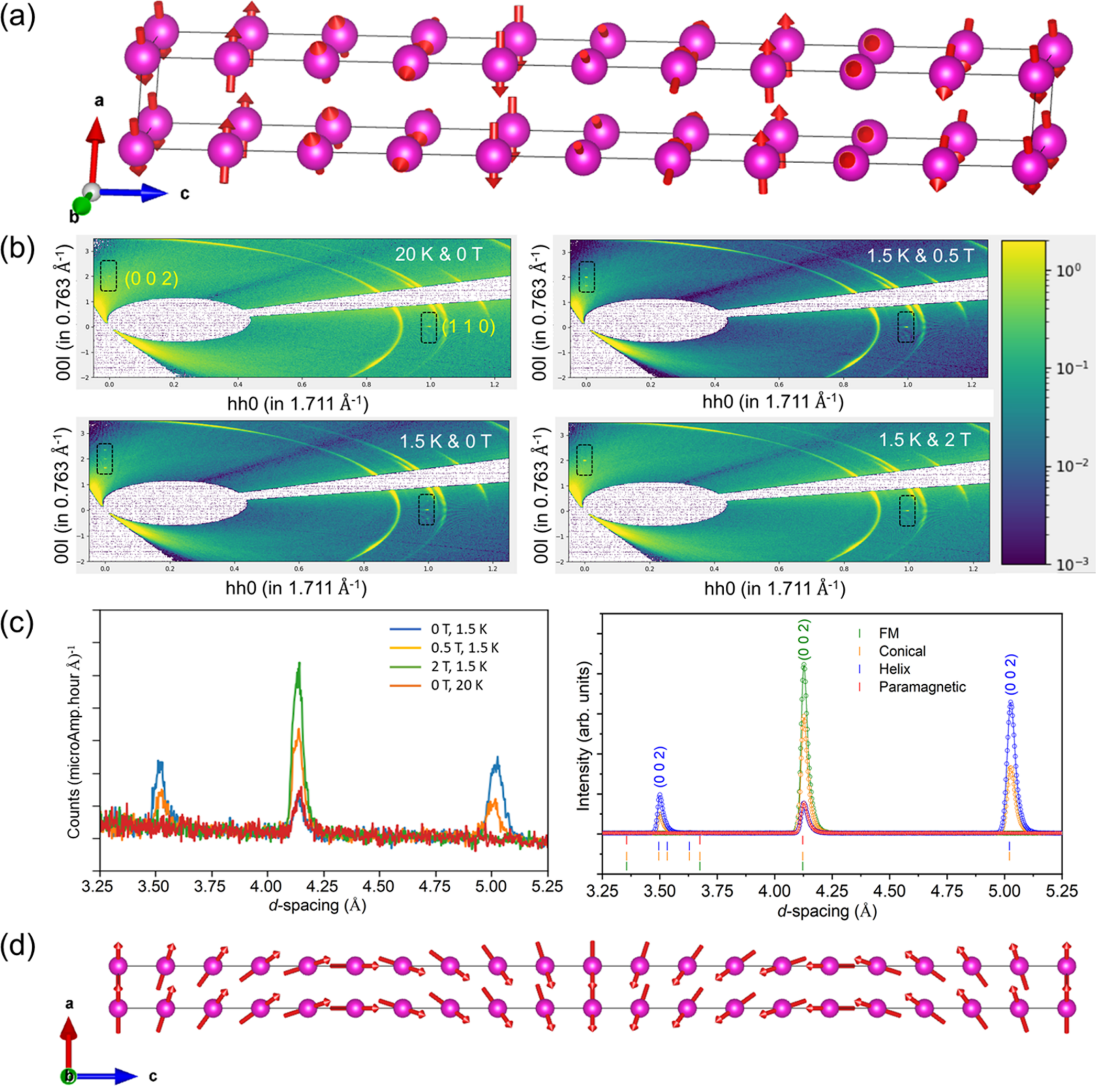
(a) Helical magnetic structure viewed along the *b*-direction. (b) Temperature and magnetic field dependent *HKL* plots obtained from the single-crystal neutron diffraction. (c) Evolution of diffraction patterns for paramagnetic, helical, conical, and ferromagnetic phases (left panel) experimentally and (right panel) simulated. (d) View of the transverse conical magnetic structure obtained from the simulation along the *b*-direction.

To develop a deeper and a clearer understanding of the magnetic spin structure, we performed powder and single-crystal neutron diffraction on EuCuAs. The refinement of the powder data confirmed that this compound crystallizes in a hexagonal structure (Figure S7a, SI). Furthermore, to reveal the ground-state magnetic structure, we collected the data at 1.5 K, which is well below *T*_N_. The appearance of new additional reflections confirms the long-range magnetic order (see Figure S7a–c, SI). Using the k_search program available in the FullProf Suite software, we found the propagation vector to be incommensurate and **k** = (0 0 *g*).^39,40^ We performed the symmetry analysis using the ISODISTORT online software and found four possible irreducible representations (irreps) corresponding to the paramagnetic space group and **k** = (0 0 *g*).^39,40^ The k-vector corresponds to the point *DT* in the Brillouin zone, and the magnetic modes, the four irreps are *mDT*2, *mDT*3, *mDT*5, and *mDT*6, which in turn have many magnetic solutions. We systematically tested all possible solutions and found that the magnetic super space group *P*6_3_22.1′(00*g*)*h*00*s* corresponding to irrep m*DT*6 is the most plausible solution. The obtained magnetic structure is shown in Figures 6a and S7d, SI, and the obtained magnetic moment is 6.95(2) μ_B_ per Eu^2+^ structural parameter. Interestingly, the deduced magnetic structure is helical, with the propagation vector (0 0 0.352(1)) along the *c*-direction and the moments rotating in the *ab*-plane.

As mentioned above, this compound exhibits an antiferromagnetic to metamagnetic transition and finally to a ferromagnetic spin structure under applied magnetic fields (Figure 1e). Interestingly, it exhibits a large topological Hall effect in the metamagnetic region (Figure 4). To study these transitions under magnetic fields and to understand the origin of the THE, we performed single-crystal neutron diffraction with and without a magnetic field. We have exposed the crystal to a neutron beam along the 00*l* direction and applied a magnetic field perpendicular to it by replicating the transport measurement configuration.

Despite a strong absorption of neutrons by the crystals, we have observed very weak satellite spots corresponding to 00*l* and *hh*0 reflections, which can be seen from the *HKL* plots provided in Figure 6b. It is clear from the figures that there are only 002 and 110 nuclear reflections in the data collected in the paramagnetic region at 20 K. At zero magnetic field and 1.5 K, two new reflections appear from the helical magnetic structure. Below 0.5 T, the pattern indicates the change in the magnetic structure, confirming the metamagnetic transition at 0.3 T, observed by physical property measurements. Furthermore, at higher magnetic fields ∼2 T, both new reflections disappear, and the intensity of the 002 nuclear reflection increases, confirming the polarized ferromagnetic state. In addition, these changes are evident in integrated intensity plots, as shown in Figure 6c.

We further simulate the diffraction patterns further for different possible solutions to obtain the spin structure above the metamagnetic transition and compare them with the experimental data. It is clear from the data at 0.5 T that we need a second **k**-vector (*k* = 0), since there is an increase in the intensity of the 002 nuclear reflection. Using FullProf, we simulate the diffraction patterns with *P*–1 and k-vectors (0 0 0.352) and (0 0 0) for cycloid, conical, and spin density wave structures which are allowed by the symmetry analysis. From Figures 6c and S8, SI, it can be seen that the best reasonable solution is obtained for a transverse conical magnetic structure.

This magnetic structure consists of a cycloid plane with a *c*-axis and a ferromagnetic component along the magnetic field, as shown in Figure 6d. The transverse conical structure is noncollinear and noncoplanar, contributing to the finite Berry phase and hence the large topological Hall effect. Finally, under high magnetic fields, the spin structure becomes ferromagnetic with **k** = 0, leading to AHE. Based on this finding, we can observe different topological states modulated by a magnetic field in EuCuAs, where its electronic structure is tightly coupled to its magnetism, as further supported by DFT calculations.

## Conclusions

In summary, we have systematically investigated the electronic structure, magnetism, and electrical transport properties of EuCuAs single crystals. EuCuAs with *T*_N_ ∼ 16 K exhibits a metamagnetic transition when μ_0_*H* ⊥ *c*, resulting in a noncoplanar spin structure in the system. The neutron diffraction experiment confirms that the helical structure of EuCuAs transforms into a noncoplanar transverse conical magnetic structure. Consequently, a large maximum of the topological Hall resistivity of ∼7.4 μΩ-cm is observed at *T* = 13 K, which is higher than that of skyrmionic and frustrated magnets. A maximum negative MR of 85% was obtained at *T* = 15 K, close to the Néel temperature. Moreover, our ARPES measurements showed reasonable agreement with the band dispersion of PM-EuCuAs calculated by DFT. By tuning the magnetic order, we discovered that multiple topological states can be obtained in a single system. The integration of magnetism and topology presented in this study is expected to open new avenues for exploring novel phenomena in the near future.

## Supporting Information Available

Experimental section, top view crystal structure, isothermal magnetization, anomalous Hall conductivity and angle, band structure, powder, and single-crystal neutron diffraction, EDXS, and single-crystal refinement parameters (PDF)
