# Supplementary material for: Interplay between Magnetism and Topology: Large Topological Hall Effect in an Antiferromagnetic Topological Insulator, EuCuAs
Source: J Am Chem Soc. 2023 Jun 2;145(23):12920–7. doi: 10.1021/jacs.3c04249 (PMC10273232; doi:10.1021/jacs.3c04249)
Supplement: Supplementary file 1 — ja3c04249_si_001.pdf [file ja3c04249_si_001.pdf]

Supporting Information (SI)

Interplay between magnetism and topology: Large topological Hall effect in antiferromagnetic topological insulator, EuCuAs

*Subhajit Roychowdhury, [1,] * Kartik Samanta, [1] Premakumar Yanda,[1] Bernard Malaman,[2] Mengyu Yao, [1] Walter Schnelle, [1] Emmanuel Guilmeau,[3] Procopios Constantinou, [4] Sushmita Chandra,[1]Horst Borrmann, [1] Maia G. Vergniory, [1,5] Vladimir Strocov, [4] Chandra Shekhar, [1] and Claudia Felser [1,] **

[1]Max Planck Institute for Chemical Physics of Solids, 01187 Dresden, Germany
[2]Institut Jean Lamour – Universit´e de Lorraine, Centre National de la Recherche Scientifique – France
[3]CRISMAT, CNRS, Normandie Univ, ENSICAEN, UNICAEN, 14000 Caen, France
[4]Swiss Light Source, Paul Scherrer Institute, CH-5232, Villigen-PSI, Switzerland
[5]Donostia International Physics Center, 20018 Donostia-San Sebastian, Spain

**Email: subhajit.roychowdhury@cpfs.mpg.de; Claudia.Felser@cpfs.mpg.de*

METHODS

Single-crystal growth of EuCuAs and characterizations

Single crystals of EuCuAs were grown from a bismuth (Bi) melt. As-purchased elemental Eu (alfa aesar, 99.9%), Cu (chemPur, 99.999%), As (alfa aesar, 99.999%), and Bi (alfa aesar, 99.999+%) pieces were mixed in the molar ratio Eu: Cu: As: Bi of 0.1:0.1:0.1:0.9 inside an Argon-filled glove box. All of the elements were loaded into an alumina crucible which was vacuum-sealed in a quartz tube under a pressure $p < 10^{-5}$ mbar. The tube was heated to 1100 ^{0}C for 15 hours, then dwelled for 24 hours before being progressively cooled to 700 ^{0}C for 267 hours. After centrifuging at 700 ^{0}C to remove excess Bi, the crystals were recovered.

The orientation of the measured single crystal was performed and crystal structure of EuCuAs was investigated using single-crystal X-ray diffraction. Refinements of room temperature X-ray diffraction data taken on a single crystal of AFM EuCuAs indicate the presence of Cu vacancy in the present system. Needle-shaped single crystals were fixed with glue on the glass capillary. The diffraction data sets were collected on a Rigaku AFC7 diffractometer equipped with a Saturn 724+ CCD detector (monochromatic MoK_{α} radiation, $\lambda = 0.71073$ Å). The intensities of the measured reflections were corrected for absorption using multi-scan technique. Structure refinement was performed by full-matrix least-squares on F within the program package WinCSD.

Electrical transport and magnetization measurements

Magnetization was measured in a MPMS3 magnetometer (Quantum Design). Further low temperature measurements were performed with an integrated magnet cryostat measurement system (PPMS9, Quantum Design). Heat capacity, c_p, was measured using the HC option of the PPMS9. The ETO option of the PPMS9 was employed to measure electrical transport properties. For these studies, the sample was cut into a rectangular bar shape and six contacts were attached using silver paint and 25μm Pt wires to measure the magnetoresistance (MR) and

Hall resistivity. The field-dependent MR and Hall resistivity data were symmetrized (antisymmetrized) to eliminate the effect of the misalignment of the electrodes.

Angle-resolved photoemission spectroscopy

Angle-resolved photoemission spectroscopy (ARPES) investigations were carried out using soft X-ray ARPES (SX-ARPES) at the ADRESS beam line of the Swiss Light Source[1] with a PHOIBOS-150 analyzer[2]. The single crystal samples were cleaved in-situ at $T = 15$ K. The base pressure was below 1×10^{-10} mbar. The data were collected using photon energies in the soft X-ray regions, with an overall energy resolution on the order of 50–80 meV.[3]

Neutron experiment

Neutron diffraction measurements on EuCuAs were carried out on the high-resolution WISH diffractometer, RAL, UK.

^{151}Eu Mössbauer experiment

The europium-151 Mössbauer spectra were measured in transmission geometry with a spectrometer operating in the conventional constant-acceleration mode that utilized a $^{151m}SmF_3$ source. Spectra were recorded at 300 K in a JANIS (Research Company Inc.) liquid helium cryostat. Polycrystalline absorbers, with natural abundance of ^{151}Eu isotope and area density of ~20 mg cm^{-2}, were used. Velocity calibration was performed against a 12-μm-thick α-Fe foil at RT using a source of ^{57}Co in Rh. The isomer shifts are reported relative to EuF_3 at room temperature. The resulting spectra were fitted with a custom Mössbauer fitting program for the ^{151}Eu (21.7 keV) resonance; a Lorentzian line profile was used in the fitting procedure.

Computational methodology

The electronic structure calculations were performed based on density functional theory (DFT) using the plane-wave projected augmented wave (PAW) method as implemented in Vienna ab initio Simulation Package (VASP)[4-6]. For the self-consistent calculations, we used a 11x11x5 k-points mesh. This choice of the k-mesh and a plane-wave cutoff of 500eV were found to provide a good convergence of the total energy. We used the Perdew-Burke-Ernzerhof (PBE)[7]

exchange correlation functional within the generalized gradient approximation (GGA*)*. The electron-electron correlation effects beyond *GGA* at the magnetic *Eu 4f* site were considered by the *GGA+U* method[8] where the value the value of the onsite Coulomb interaction strength *U* chosen to be 5 eV. Employing the Wannier interpolation technique[9] we assess the intrinsic Berry curvature contribution to the anomalous Hall conductivity. To compute the Berry curvature, we first constructed a tight-binding maximally localized Wannier functions (MLWFs) Hamiltonian projected from the *GGA+U+SOC* Bloch wave functions using the Wannier90 tool[10-12]. Atomic orbital-like MLWFs of Eu-*s, d, f ,* Cu-*s, p, d* and As-*p* states are considered to construct the tight-binding Hamiltonian, which reproduces the spectrum of the system accurately in the energy window of ± 2 eV around the Fermi energy. With the tight-binding model Hamiltonian, we calculated the intrinsic AHC using the linear response Kubo formula approach[13]. We used a *k*-point mesh of 300×300×300 for the calculation of the AHC. We used the FINDSYM tool to understand the magnetic space group and symmetry for different magnetic configurations.[14]

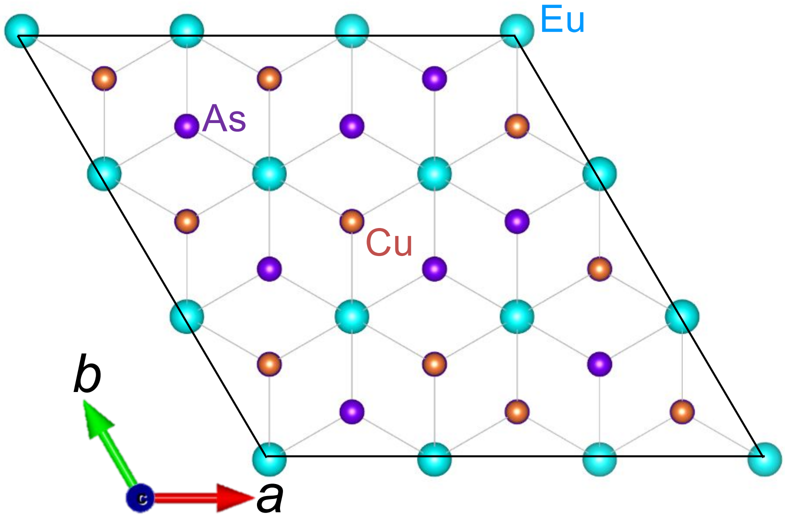

Figure S1: Top view projection (along the *c*-axis) of the crystallographic unit cell of EuCuAs.

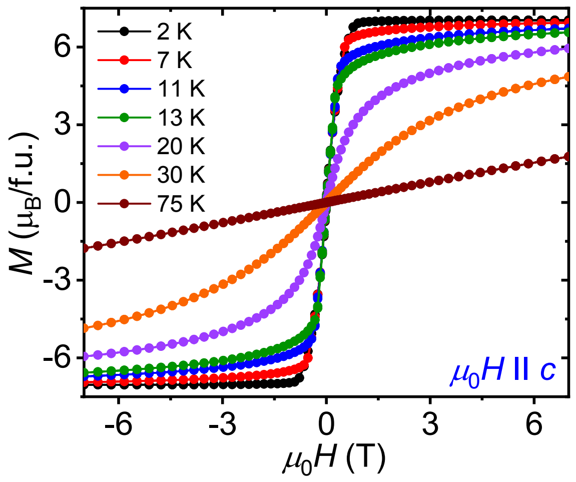

Figure S2: Isothermal magnetization for $\mu_0 H \parallel c$ at several temperatures.

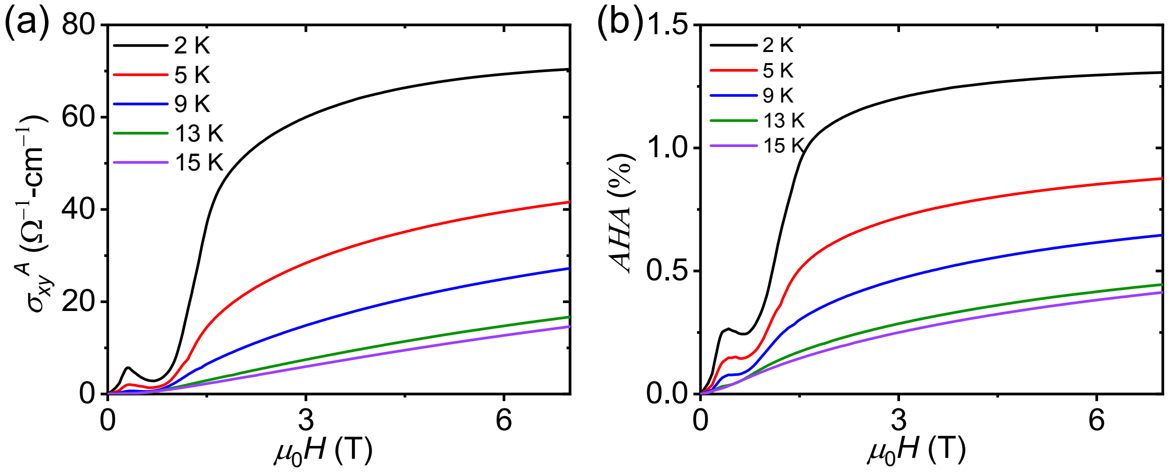

Figure S3: Field-dependence of the (a) anomalous Hall conductivity, $\sigma_{xy}{}^{A}$, and (b) anomalous Hall angle (AHA ~ $\sigma_{xy}{}^{A}/\ \sigma_{xx}$) of EuCuAs at different temperatures.

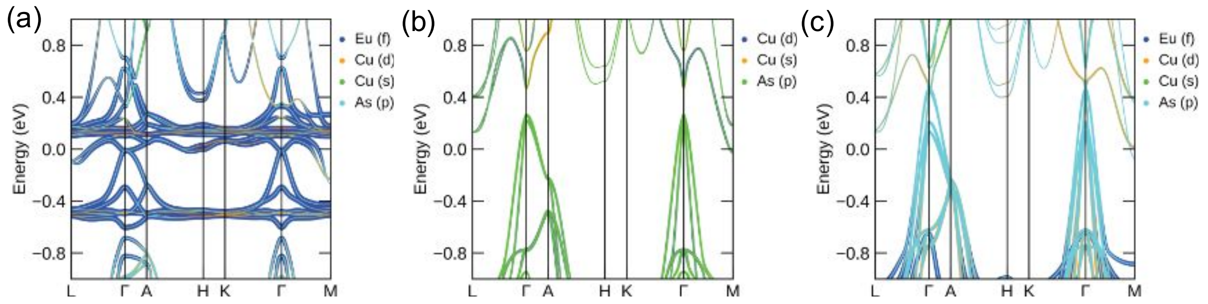

Figure S4: Site projected electronic band structure of (a) paramagnetic (b) antiferromagnetic and (c) ferromagnetic phase of EuCuAs.

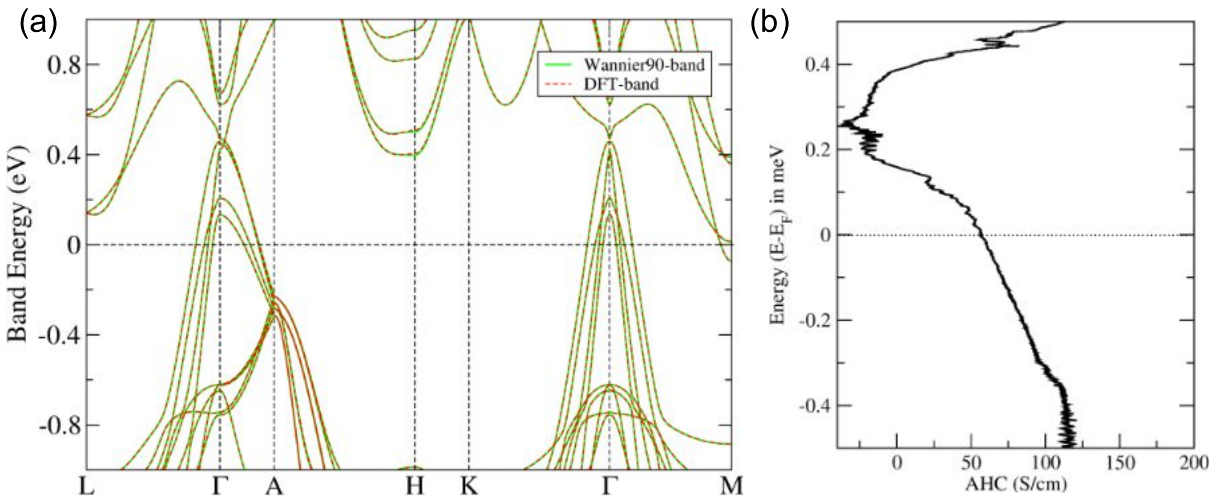

Figure S5: (a) Electronic Band structure of EuCuAs in *GGA + U + SOC* for the FM state with magnetic moment along the *c* axis. Green lines: *GGA + U + SOC* first principles electronic bands. Red lines: Wannier-interpolated band structure. (b) Theoretically calculated anomalous Hall conductivity (AHC) as a function of the chemical potential.

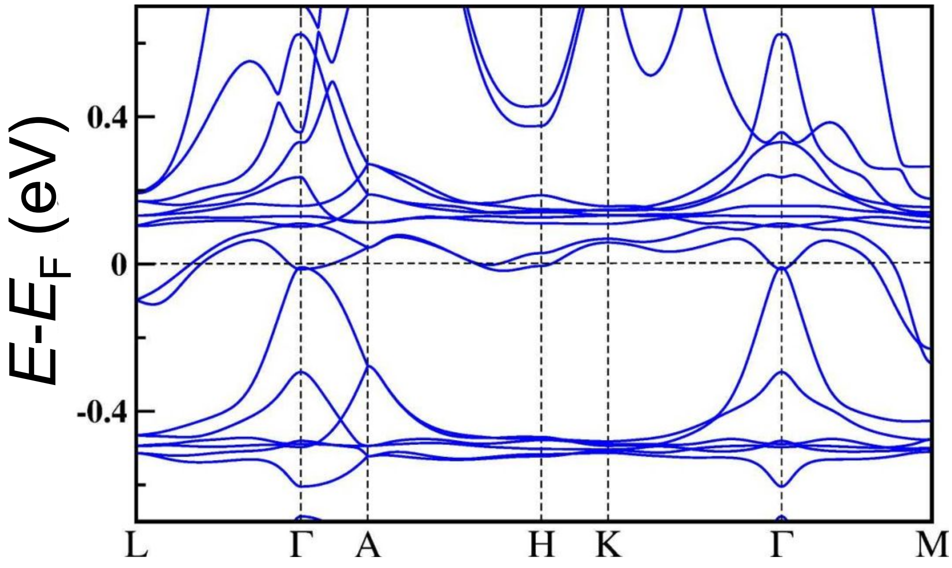

Figure S6: Electronic band structure of paramagnetic EuCuAs.

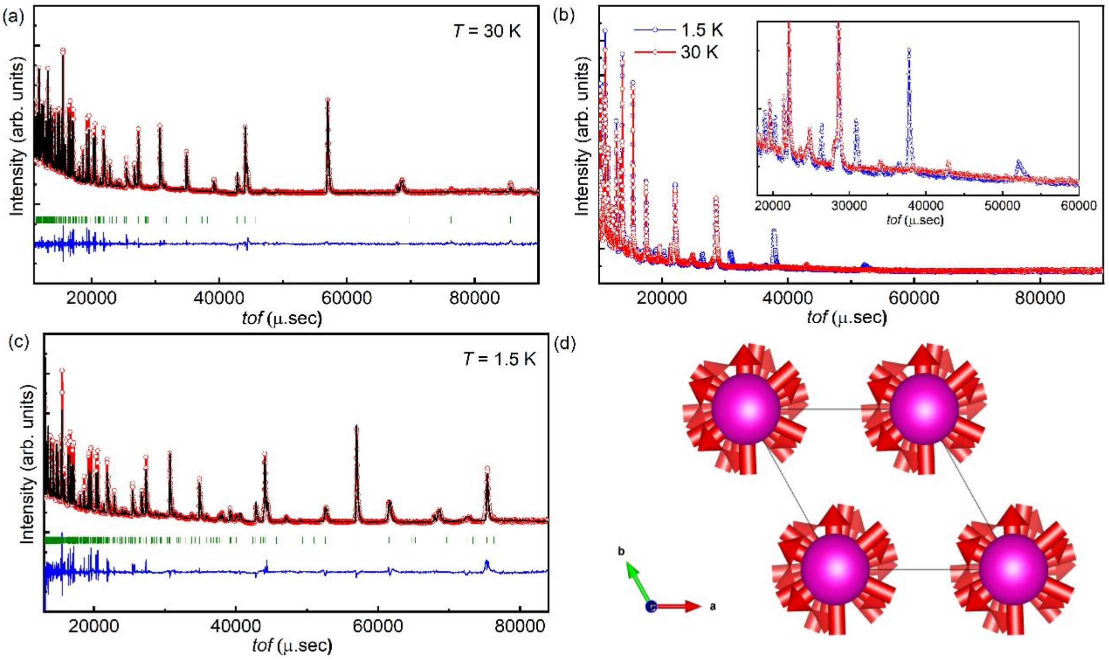

Figure S7: Rietveld refinement of powder neutron diffraction data collected at (a) 30 K and (b) 1.5 K. (c) Comparison between the neutron diffraction data at 1.5 and 30 K. (d) Helical magnetic structure viewed along *c*-direction.

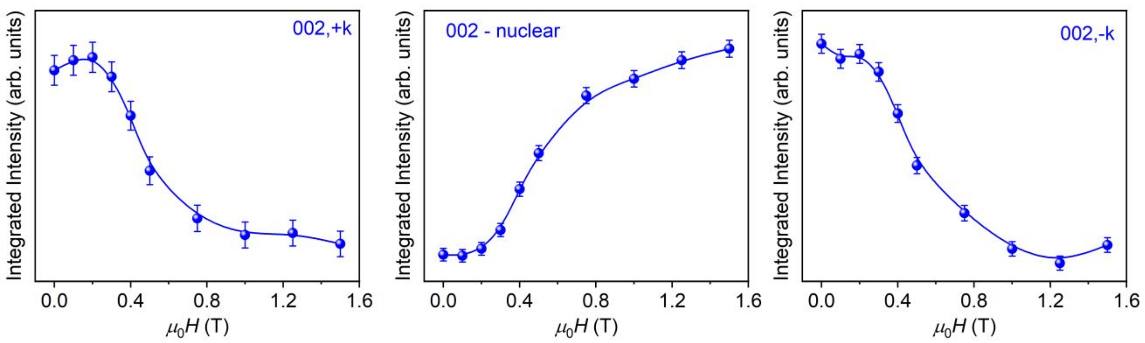

Figure S8: Integrated intensity for the 002 nuclear reflection and the corresponding magnetic reflections.

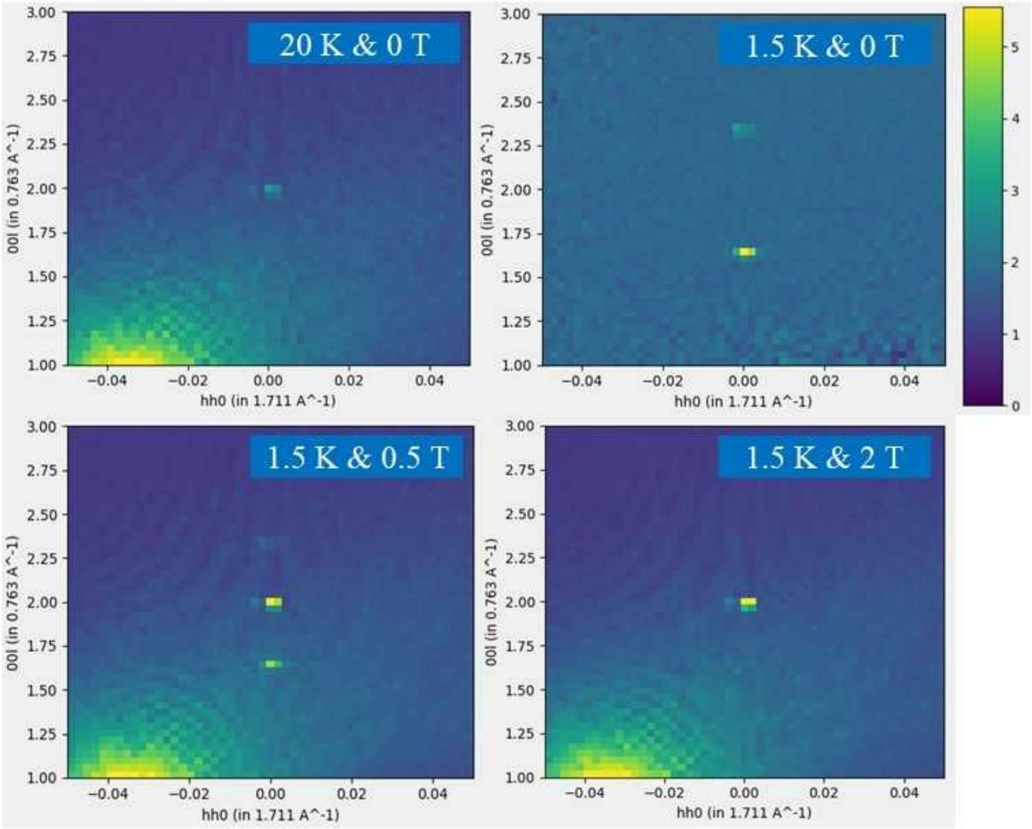

Figure S9: Temperature and magnetic field dependent HKL plots obtained from the single crystal neutron diffraction.

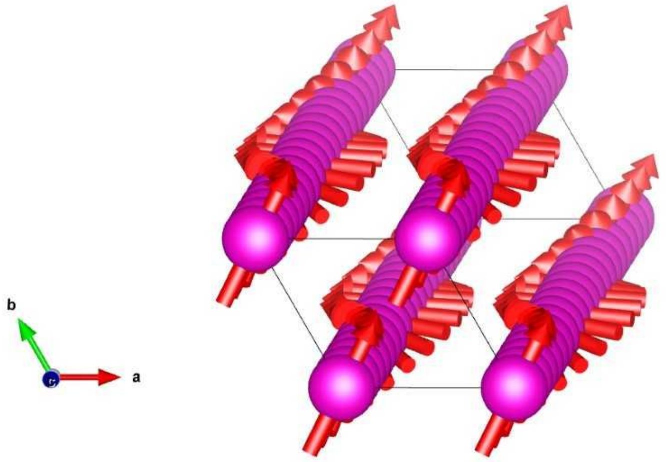

Figure S10: View of transverse conical magnetic structure obtained from simulation along *c*-directions.

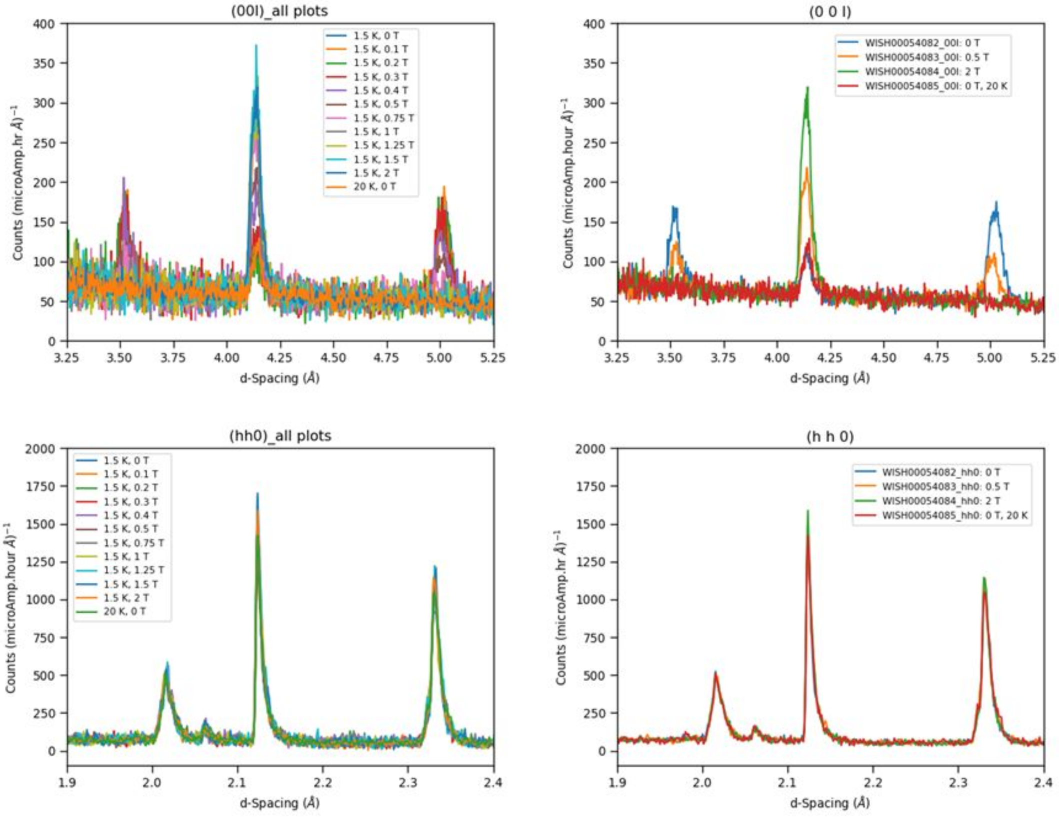

Figure S11: Evolution of diffraction patterns of *00l* and *hh0* reflections for paramagnetic, helical, conical, and ferromagnetic phases (left panel) experimental and (right panel) simulated.

Table S1: Composition of the EuCuAs crystal determined by Energy Dispersive X-ray Spectroscopy (EDXS).

Element	Wt%	At%
Eu	54.23	35.08
Cu	20.72	32.05
As	25.05	32.87

TABLE S2: Parameters determined from refinements of room temperature X-ray diffraction data taken on a single crystal of AFM EuCuAs.

Parameters	AFM- EuCuAs
a (Å)	4.2598(3)
c (Å)	8.2857(9)
V (Å^3)	130.21(3)
space group	*P*6$_3$/*mmc* (no. 194)
Eu site occupancy	1.008(3)
Cu site occupancy	0.976(5)
As site occupancy	1.001(3)
U_{Eu} (Å^2)	0.00830(11)
U_{Cu}(Å^2)	0.00900(18)
U_{As}(Å^2)	0.00706(12)
Reflections collected	4216
Unique Reflections	270
Refined Parameters	9
Goodness-of-fit	2.564
R[F][a]	0.0254
$R_w(F_0^2)$[b]	0.0545

[a]$R(F) = \Sigma||F_o| - |F_c||/\Sigma|F_o|$

[b]$R_w(F_0^2) = [\Sigma_w(F_0^2 – F_c^2)2 /\Sigma_w(F_0^2)2]^{1/2}$

CCDC-2259717 contains a full set of crystallographic data for this structure. The data can be obtained free of charge from The Cambridge Crystallographic Data Centre via www.ccdc.cam.ac.uk/structures.

References

1. Strocov, V. N.; Schmitt, T.; Flechsig, U.; Schmidt, T.; Imhof, A.; Chen, Q.; Raabe, J.; Betemps, R.; Zimoch, D.; Krempasky, J.; Wang, X.; Grioni, M.; Piazzalunga, A.; Patthey, L. High-resolution soft X-ray beamline ADRESS at the Swiss Light Source for resonant inelastic X-ray scattering and angle-resolved photoelectron spectroscopies. *J. Synchrotron Rad.* **2010**, *17*, 631.
2. Strocov, V. N.; Wang, X.; Shi, M.; Kobayashi, M.; Krempasky, J.; Hess, C.; Schmitt, T.; Patthey, L. Soft-X-ray ARPES facility at the ADRESS beamline of the SLS: concepts, technical realisation and scientific applications. *J. Synchrotron Radiat.* **2014**, *21*, 32.
3. Strocov, V. N.; Shi, M.; Kobayashi, M.; Monney, C.; Wang, X.; Krempasky, J.; Schmitt, T.; Patthey, L.; Berger, H.; Blaha, P. Three-Dimensional Electron Realm in VSe2 by Soft-X-Ray Photoelectron Spectroscopy: Origin of Charge-Density Waves. *Phys. Rev. Lett.* **2012**, *109*, 086401.
4. Kresse, G.; Furthmuller, J. Efficient Iterative Schemes for Ab initio Total-Energy Calculations Using a Plane-Wave Basis Set. *Phys. Rev B* **1996**, *54*, 11169.
5. Kresse, G.; Joubert, D. From ultrasoft pseudopotentials to the projector augmented-wave method. *Phys. Rev. B* **1999**, *59*, 1758.
6. Blöchl, P. E. Projector augmented-wave method. *Phys. Rev. B* **1994**, *50*, 17953.
7. Perdew, J. P.; Burke, K.; Ernzerhof, M. Generalized gradient approximation made simple. *Phys. Rev. Lett.* **1996**, *77*, 3865.
8. Anisimov, V. I.; Aryasetiawan, F.; Lichtenstein, A. I. First principles calculations of the electronic structure and spectra of strongly correlated systems: the LDA+ U method, *J. Phys.: Condens. Matter.* **1997**, *9* ,767.
9. Wang, X.; Yates, J. R.; Souza, I.; Vanderbilt, D. Ab initio calculation of the anomalous Hall conductivity by Wannier interpolation. *Phys. Rev. B* **2006**, *74*, 195118.
10. Marzari, N.; Mostofi, A. A.; Yates, J. R.; Souza, I.; Vanderbilt, D. Maximally localized Wannier functions: Theory and applications. *Rev. Mod. Phys*. **2012**, *84*, 1419.
11. Souza, I.; Marzari, N.; Vanderbilt, D. Maximally localized Wannier functions for entangled energy bands. *Phys. Rev. B* **2001**, *65*, 035109.
12. Mostofi, A. A. et al. An updated version of wannier90: A tool for obtaining maximally-localised Wannier functions. *Comput. Phys. Commun*. **2014**, *185*, 2309.
13. Yao, Y.; Kleinman, L.; MacDonald, A. H.; Sinova, J.; Jungwirth, T.; Wang, D.; Wang, E.; Niu, Q. First Principles Calculation of Anomalous Hall Conductivity in Ferromagnetic bcc Fe. *Phys. Rev. Lett*. **2004**, *92*, 037204.
14. Stokes, H. T.; Hatch, D. M. Program for identifying the space group symmetry of a crystal. *J. Appl. Cryst*. **2005**, *38*, 237.
